# Supplementary material for: Notched Belly Grain 4, a Novel Allele of Dwarf 11, Regulates Grain Shape and Seed Germination in Rice (Oryza sativa L.)
Source: Int J Mol Sci. 2018 Dec 16;19(12):4069. doi: 10.3390/ijms19124069 (PMC6321642; doi:10.3390/ijms19124069)
Supplement: Supplementary file 1 [file ijms-19-04069-s001.zip › Supplementary File/ijms-392953-supplmentary figure S1 and S2 (2).docx]

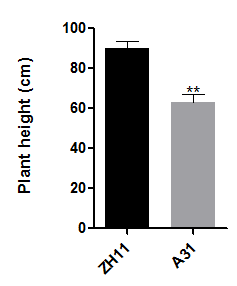

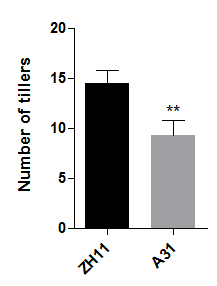


**A B**

**Figure S1.** Plant height and tiller numbers of A31 and ZH11.

Numerical values are expressed as the mean; error bars denote one standard deviation of the mean; asterisk denotes significant difference between groups (**p* < 0.05,***p* < 0.01; Student’s *t*-test)


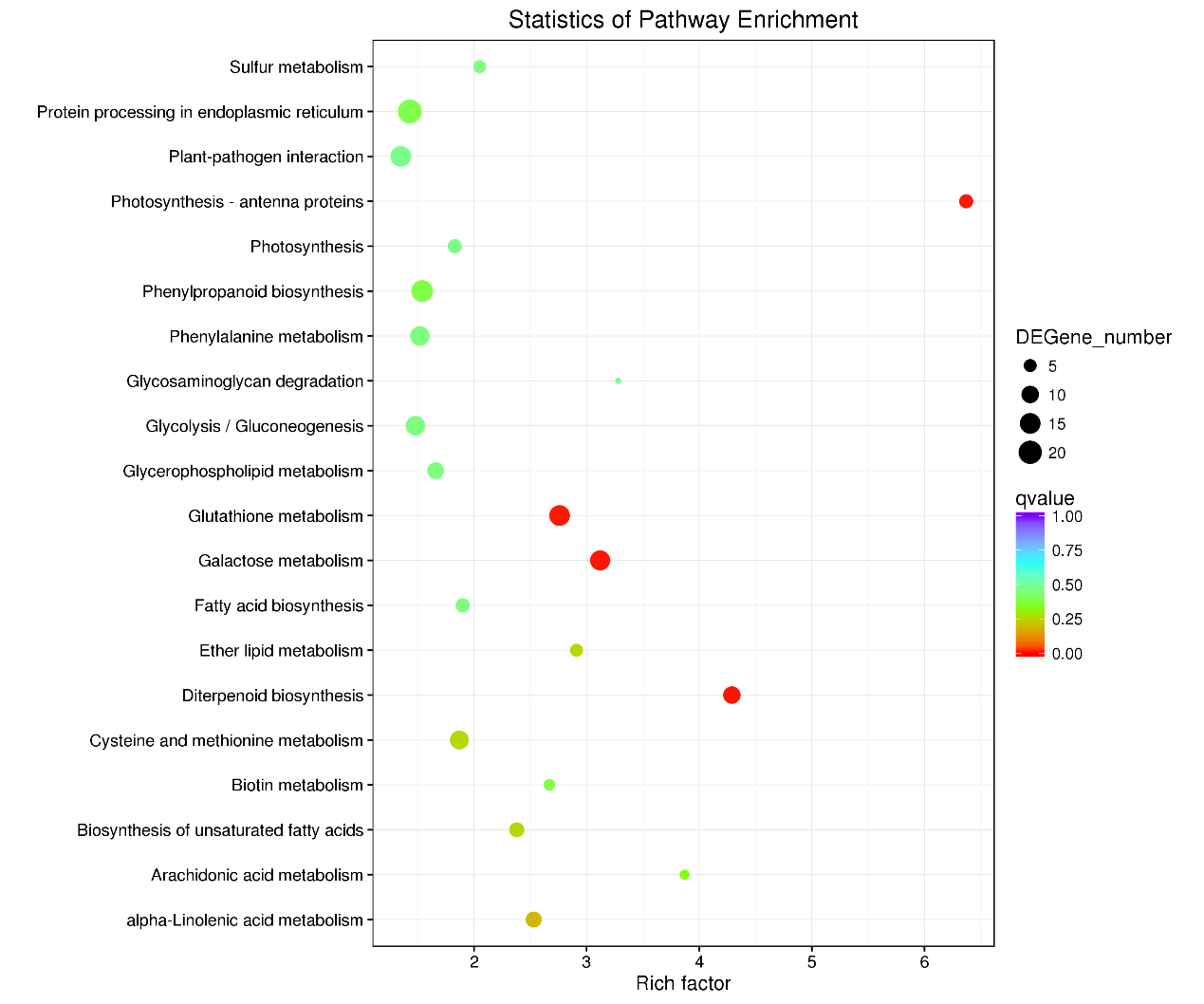


**Figure S2.** KEGG pathway enrichment assay of DEGs.
